# Murine Efficacy and Pharmacokinetic Evaluation of the Flaviviral NS5 Capping Enzyme 2-Thioxothiazolidin-4-One Inhibitor BG-323

**DOI:** 10.1371/journal.pone.0130083

**Published:** 2015-06-15

**Authors:** Kristen M. Bullard, Rebekah C. Gullberg, Elnaz Soltani, J. Jordan Steel, Brian J. Geiss, Susan M. Keenan

**Affiliations:** 1 University of Northern Colorado, School of Biological Sciences, Greeley, CO, United States of America; 2 Department of Microbiology, Immunology, and Pathology, Colorado State University, Fort Collins, Colorado, United States of America; 3 Department of Biochemistry and Molecular Biology, Colorado State University, Fort Collins, Colorado, United States of America; Washington University, UNITED STATES

## Abstract

Arthropod-borne flavivirus infection continues to cause significant morbidity and mortality worldwide. Identification of drug targets and novel antiflaviviral compounds to treat these diseases has become a global health imperative. A previous screen of 235,456 commercially available small molecules identified the 2-thioxothiazolidin-4-one family of compounds as inhibitors of the flaviviral NS5 capping enzyme, a promising target for antiviral drug development. Rational drug design methodologies enabled identification of lead compound BG-323 from this series. We have shown previously that BG-323 potently inhibits NS5 capping enzyme activity, displays antiviral effects in dengue virus replicon assays and inhibits growth of West Nile and yellow fever viruses with low cytotoxicity *in vitro*. In this study we further characterized BG-323’s antiviral activity *in vitro* and *in vivo*. We found that BG-323 was able to reduce replication of WNV (NY99) and Powassan viruses in culture, and we were unable to force resistance into WNV (Kunjin) in long-term culture experiments. We then evaluated the antiviral activity of BG-323 in a murine model. Mice were challenged with WNV NY99 and administered BG-323 or mock by IP inoculation immediately post challenge and twice daily thereafter. Mice were bled and viremia was quantified on day three. No significant differences in viremia were observed between BG-323-treated and control groups and clinical scores indicated both BG-323-treated and control mice developed signs of illness on approximately the same day post challenge. To determine whether differences in *in vitro* and *in vivo* efficacy were due to unfavorable pharmacokinetic properties of BG-323, we conducted a pharmacokinetic evaluation of this small molecule. Insights from pharmacokinetic studies indicate that BG-323 is cell permeable, has a low efflux ratio and does not significantly inhibit two common cytochrome P450 (CYP P450) isoforms thus suggesting this molecule may be less likely to cause adverse drug interactions. However, the T_1/2_ of BG-323 was suboptimal and the percent of drug bound to plasma binding proteins was high. Future studies with BG-323 will be aimed at increasing the T_1/2_ and determining strategies for mitigating the effects of high plasma protein binding, which likely contribute to low *in vivo* efficacy.

## Introduction

Diseases caused by infection with arthropod-borne flaviviruses such as those resulting from infection by dengue virus, yellow fever virus and West Nile virus (WNV) continue to plague populations worldwide. The World Health Organization estimates that almost half the global population is at risk of dengue virus infection and 900 million people live in areas endemic for yellow fever transmission [1]. Each year there are an estimated 200,000 cases of yellow fever and 400 million cases of dengue fever leading to ~30,000 and ~20,000 deaths respectively [2]; and alarmingly, flavivirus transmission rates have continued to rise over the last two decades. Currently, there are no effective treatments for diseases caused by flavivirus infections. Thus, there is an immediate need to validate anti-flaviviral drug targets and identify compounds with the ability to inhibit flaviviral replication.

Flaviviruses such as yellow fever, dengue, and West Nile viruses reside in the family *Flaviviridae* and the genus *Flavivirus* along with approximately 70 other known human pathogens [3]. The flavivirus genome consists of 10.7–11 kb positive-sense single-stranded RNA with a 5’ type 1 RNA cap, which prevents degradation of the viral genome and is necessary for translation initiation [4,5]. The flavivirus genome codes for a single polyprotein precursor that is eventually cleaved by host and viral proteases into three structural proteins (C, prM and E) and eight nonstructural proteins (NS1, NS2A, NS2B, NS3, NS4A, 2K, NS4B and NS5) [6]. While structural proteins contribute to formation of the mature virion, nonstructural proteins carry out replication of the viral genome and protect the replicating virus from attack by the host’s immune system by modulating the host cell environment [6]. Of the 11 virus proteins, four have been identified as promising targets for antiviral drug development including the multifunctional NS5 protein, which possesses RNA dependent RNA polymerase, methyltransferase (MTase) and guanylyltransferase (GTase) activities (reviewed in [7]).

The N-terminal capping enzyme domain of the NS5 protein in particular shows promise as a point of therapeutic intervention. This domain is responsible not only for binding GTP, but it also orchestrates the N7-MTase, 2′O-MTase and RNA GTase activities necessary for cap formation [8,9,10,11]. It has been shown that mutation of residues within the DEN capping enzyme domain eliminates viral replication, thus highlighting the essential nature of its functions [10,12,13,14]. Additionally, evidence suggests that it may be possible to selectively target the GTP-binding activity of the NS5 capping enzyme, therefore reducing the likelihood of undesirable drug effects [7,15]. Studies have shown that the viral enzyme binds GTP in a manner distinct from host cell GTP-binding proteins [16,17,18,19,20,21]. Further, the high degree of structural conservation observed among crystal structures from multiple flavivirus capping enzymes suggests that this unique binding mechanism is preserved among all known flaviviral capping enzymes and capping enzyme-targeted inhibitors may have broad spectrum anti-flaviviral applications [7,16,21,22,23]. Taken together, the necessity of capping enzyme activity for viral replication, the unique nature GTP binding observed in the NS5 capping enzyme, and the potential broad spectrum applications of flavivirus capping enzyme inhibitors make the capping enzyme an attractive target for antiviral drug design.

Previously, we developed a robust fluorescence polarization (FP) assay to monitor NS5 capping enzyme GTP-binding activity and screened small molecules in real time. We adapted this assay for high-throughput screening [21] and conducted a pilot screen of 46,323 small molecules [24]. More recently, we used this assay to screen 235,456 compounds from the National Screening Laboratory for the Regional Centers of Excellence in Biodefense (NSRB) library at the Harvard Medical School Longwood Campus. As a result of these efforts, we identified several families of compounds that were able to competitively inhibit GTP binding to the capping enzyme. The 2-thioxothiazolidin-4-one family was identified as a promising core because in addition to potent inhibition of capping enzyme-GTP binding, the thioxothiazolidin moiety is found in FDA-approved drugs such as Epalrestat suggesting that this core would be amenable to drug development. Structure-activity relationship (SAR) analysis of analogs from the 2-thioxothiazolidin-4-one core identified (E)-{3-[5-94-tert-butylbenzylidene0-4-oxo-2-thioxo-1,3-thiazolidin-3-yl] propanoic acid} (also known as BG-323) as a lead compound [15]. BG-323 is able to displace GTP in the capping enzyme GTP-binding site, inhibit guanylation activity in an orthogonal *in vitro* assay, and prevent viral replication of several flaviviruses in cell culture [15]. Further, BG-323 was also shown to have low cytotoxicity and a relatively high therapeutic index as compared to the best known antiflaviviral drug, ribavirin.

Here, we report on the continued investigation of the NS5 capping enzyme inhibitor BG-323 as a potential anti-flaviviral agent. We first examined the *in vitro* efficacy of BG-323 and found that it was able to inhibit virulent WNV (NY99 strain) and tick-borne Powassan virus replication. We then assessed the ability of WNV (Kunjin) to develop BG-323 resistance and found that Kunjin virus did not display any signs of BG-323 resistance following continuous BG-323 administration. We next evaluated the *in vivo* efficacy of BG-323. BG-323 elicits no signs of gross toxicity in mice; however, our findings also indicate little difference in viral titers or clinical scores between BG-323-treated and control West Nile virus-challenged mice. To better understand the lack of *in vivo* efficacy, we also conducted a pharmacokinetic evaluation of the compound. The half-life (T_1/2_) of BG-323 in mice was found to be suboptimal and other *in vitro* analyses indicate that BG-323 is cell permeable, has a low efflux ratio, and does not activate two CYP P450 isoforms. Finally, our data indicate that BG-323 binds significantly to human plasma binding proteins, which along with a relatively short half-life may account for low *in vivo* efficacy.

## Materials and Methods

### Cell culture

Baby Hamster Kidney (BHK) and Huh7 cells were maintained in Hyclone DMEM supplemented with 10% fetal bovine serum (FBS), 5% Pen/Strep, and 5% L-Glutamine. BHK and Huh7 cells were grown in a 37°C incubator with 5% CO2. West Nile virus (Kunjin subtype) was generously provided by Alexander Khromykh (University of Queensland), West Nile virus (NY99 strain) was obtained from Rich Kinney at the Centers for Disease Control (Fort Collins, CO) [25], and Powassan virus (LB strain) was provided by Dr. Greg Ebel (Colorado State University).

### *In vitro* West Nile virus and Powassan virus efficacy assays

BHK 21 cells (ATCC) were plated into six-well plates at 100,000 cells/well and allowed to attach overnight. The following day, cells were infected with 0.01 multiplicity of infection (MOI) of either West Nile or Powassan viruses and DMSO or 75 μM BG-323. Samples were collected 72 hr post infection and viral titers were determined by plaque assay as previously described [15].

### BG-323 resistance study

On day one, BHK-21 cells (ATCC) were seeded in 12-well plates at 50,000 cells/mL and allowed to attach overnight. The following day, each of 9 wells was infected with 100 PFU WNV (Kunjin subtype) in complete DMEM (cDMEM) and 3 uninfected control wells received cDMEM only. Cells were incubated with media for 2 hr at 37°C to allow cells to become infected. After 2 hr, infected cDMEM was replaced with new media containing DMSO or 75 μM BG-323. Each condition was run in triplicate. Following DMSO or drug administration, cells were incubated at 37°C. On day 3, samples were collected and stored at -80°C. Viral titers were calculated by plaque assay as previously described [24] on one tube of the day 3 samples for each replicate. 100 PFU from an unthawed day 3 sample was used to infect fresh BHK 21 cells to start the next round of infection. The three-day cycle was considered one round of infection and the study continued for 6 rounds. The infectious input of Kunjin virus and the concentrations (75 μM) of BG-323 were held constant throughout the study.

### Animal studies

Animals used in toxicity, efficacy and half-life studies were 6–8 week old, female ICR mice. Research on animals in the following studies complied with relevant federal guidelines and all protocols requiring animal use were reviewed and approved by the Colorado State University Institutional Animal Care and Use Committee (IACUC), protocol 12-3611A.

### *In vivo* toxicity assay

Two groups of 3 mice each were either injected with 150 μL of 50 mg/kg BG-323 (synthesized by Brian McNaughton’s group at CSU [15]) formulated in 10% DMSO, 15% solutol and 75% saline or DMSO vehicle in the same formulation. Mice were weighed daily for 7 days and percent of maximum weight loss was calculated.

### *In vivo* antiviral efficacy

Experiments with WNV NY99 were performed in a BSL-3 suite at Colorado State University. Two groups of 10 mice each were challenged with 500 PFU of WNV via footpad injection. Immediately post-challenge, one group was treated with 50 mg/kg of BG-323 formulated in 10% DMSO, 15% solutol and 75% saline (150 μL total volume) and one group received only DMSO vehicle in the same formulation. Mice were given doses of BG-323 or vehicle BID (bi-daily) for 5 days in the previously mentioned formulation, which was prepared daily. Mice were bled on day 3 post challenge, blood was centrifuged and resulting sera were frozen immediately at -80°C. Viremia was quantified by plaque assay as previously described [15] and clinical scores were assigned to all mice up to 14 days post infection. Mice were monitored daily for clinical signs of morbidity, including fur ruffling, hunching, and lack of responsiveness to gentle prodding. Three animals died due to WNV infection; however, moribund animals were euthanized when they exhibited severe signs of disease.

### *In vivo* half-life (T_1/2_)

A group of 13 mice were weighed and then dosed with 380 μL of BG-323 (5 mg/mL in 10% DMSO, 15% solutol (Sigma-Aldrich) and 75% saline). Ketamine-xylazine was used to anesthetize mice and mice were then bled by cardiac puncture. Three mice were bled at each of the following times; 5 min, 30 min and 120 min. Four mice were bled at 360 min. Bleeds were centrifuged and resulting sera were frozen no longer than 30 min from the time of collection. BG-323 concentration in each serum sample was determined by LC/MS/MS, which was performed at the Colorado State University Animal Cancer Center. Standards of BG-323 were prepared by collecting blank mouse plasma and spiking it with BG-323 to create a standard curve. Following sample preparations, 50 μL standard or sample was spiked with 5 μL of 2500 ng/mL (final concentration 250 ng/mL) Naringenin (Sigma-Aldrich) as an internal standard. Aliquots were extracted with 100 μL acetonitrile and vortexed for 10 min. Samples were then centrifuged for 10 min at 13,300 rpm and transferred to auto-sampler vials with inserts.

Samples were analyzed using a Waters XBridge Phenyl 5 μM, 4.6 x 50 mm column (Part No. 186003349) with a Phenomenex C18 Filter Frit Guard Cartridge on the Shimadzu HPLC system coupled to the 3200 Q-TRAP triple quadrupole mass spectrometer (Applied Biosystems, INC., Foster City, CA). Injection volume was 5 μL on a 20 μL loop using a flow rate of 1100 μL /min over a run time of 5 min. Mass spectrometry conditions were as follows: 1 min 30% methanol / 70% 10 mM ammonium acetate; 2.25 min 98% methanol / 2% 10 mM ammonium acetate; 4.0 min 98% methanol / 2% 10 mM ammonium acetate; 4.5 min 30% methanol / 70% 10 mM ammonium acetate; 5 min 30% methanol / 70% 10 mM ammonium acetate.

Mass spectrometer settings for BG-323 and Naringenin were as follows: spray temperature, 575°C; ion spray voltage, -4500; declustering potential (DP), -26.00 (BG-323) and -36.58 (Naringenin); entrance potential (EP), -4.55 (BG-323) and -3.37 (Naringenin); collision energy (CE), -14.2 (BG-323) and -39.51 (Naringenin); collision cell entrance potential (CEP), -11.28 (BG-323) and -41.20 (Naringenin); collision cell exit potential (CXP), -3.19 (BG-323) and -1.19 (Naringenin). Unknown samples were quantified using the internal standard reference method in MRM negative ion mode. Monitored ion transitions *m/z* were 348.0–276.1 amu for BG-323 and 271.0–119.0 amu for Naringenin. Ion transitions were monitored every 500 ms and unit resolution mode was used for Q1 and Q3. BG-323 concentrations in each sample were quantitated using linear standard curves as previously described [26].

### *In vitro* pharmacokinetic studies

Caco-2 cell permeability, metabolic stability, CYP P450 binding and human plasma protein binding assays were conducted by Absorption Systems (Exton, PA USA).

### Caco-2 cell permeability assay

BG-323 stock in DMSO was diluted to a final concentration of 5 μM in Hanks Buffered Salt Solution (HBSS) with 1% DMSO. The assay system was a Transwell system containing confluent monolayers of 21-28-day-old Caco-2 cells with 1% bovine serum albumin in HBSS in the receiver well and both apical and basolateral sides at pH 7.4. Monolayer quality was tested using an internal control prior to use by verifying trans-epithelial electrical resistance measurements and calculating the apparent permeability coefficient (P_app_) for the control compound prior to monolayer use. Each side of the monolayer, apical for A to B assessment and basolateral for B to A assessment, was dosed twice and resulting flow through was sampled after 120 min. Influx and efflux of BG-323 was quantified using LC-MS/MS with a minimum 4 point standard curve. Percent recovery and the P_app_ were determined for BG-323 in each direction. Efflux ratio (P_app_ B to A) / (P_app_ A to B) was also calculated. Cell permeability is considered low when the P_app_ is < 1.0 x 10^−6^ cm/s and high when A_pp_ is ≥ 1.0 x 10^−6^ cm/s^.^ Significant efflux occurs when the efflux ratio is ≥ 3 and P_app_ (B to A) ≥ 1.0 x 10^−6^ cm/s.

### Metabolic stability assay

BG-323 was diluted to 1 μM in 0.25% DMSO. Pooled liver microsomes (XenoTech) from mixed-gender donors were isolated. BG-323 was incubated in buffer containing 0.5 mg/mL microsomal protein. After initiating the reaction by adding 1 mM NADPH, samples were taken at 0, 10, 20, 30 and 60 min post-initiation. Testosterone (5 μM final concentration) was used as a positive control to verify enzymatic activity of microsomes and was sampled in parallel at 0, 10, 30 and 60 min post-initiation. After 60 min, fluorometry was used to verify that NADPH has been added to the reaction mixture. LC-MS/MS was used to calculate the peak response ratio, which is the peak area of the test compound or control divided by the peak area of the analytical standard.

### CYP P450 binding assay

Assays were run in duplicate for two CYP P450 isoforms, 2C9 and 3A4. Mixed-gender, pooled, human liver microsomes (XenoTech) were prepared from a minimum of 10 donors. BG-323 (10 μM final concentration) was incubated with 0.25 mg/mL microsomal protein at 37°C in phosphate buffer (100 mM NaPO_4_, 5 mM MgCl_2_, pH 7.4) Diclofenac and Midazolam for 2C9 and 3A4 respectively, were added at the K_m_ concentration for the enzyme. Each assay contained a maximum activity control, a single concentration of BG-323 and a positive control inhibitor. Positive control inhibitor for 2C9 was sulfaphenazole and positive control inhibitor for 3A4 was ketoconazole. Reactions were initiated by the addition of 1 mM NADPH. CYP P450 isoform 2C9 and 3A4 substrate metabolites, 4’-OH diclofenac and 1’-OH midazolam respectively, were detected using LC-MS/MS. Degree of CYP binding was calculated by determining the peak area of each CYP-specific probe substrate metabolite divided by the peak area of the analytical internal standard thus yielding a peak area response ratio (PARR). Percent inhibition was calculated as follows: % inhibition = [(PARR without test compound–PARR with test compound) / (PARR without test compound)] x 100.

### Plasma protein binding assay

BG-323 was diluted in human plasma at pH 7.4 with 1% DMSO. Human plasma (Bioreclamation) was derived from 3 or more mixed gender donors. BG-323 was tested in duplicate at a final concentration of 5 μM and dialysis took place in a Rapid Equilibrium Dialysis device (Pierce Biotechnology, Inc.). One side of each dialysis well contained human plasma and the other side contained PBS. Solutions on both sides were supplemented with sodium heparin and kept at pH 7.4. The system was allowed to equilibrate to 37°C and samples were taken four hr after temperature equilibration. LC-MS/MS was used to calculate the test compound peak area, which was then divided by the peak area of the internal standard to yield a PARR. Percent of BG-323 bound to human plasma binding protein was calculated as follows: % bound = [PARR (donor)]–[PARR (receiver)] x 100] / [PARR (donor)].

## Results

### Antiviral efficacy of BG-323 against virulent WNV and Powassan viruses

**Fig 1:**
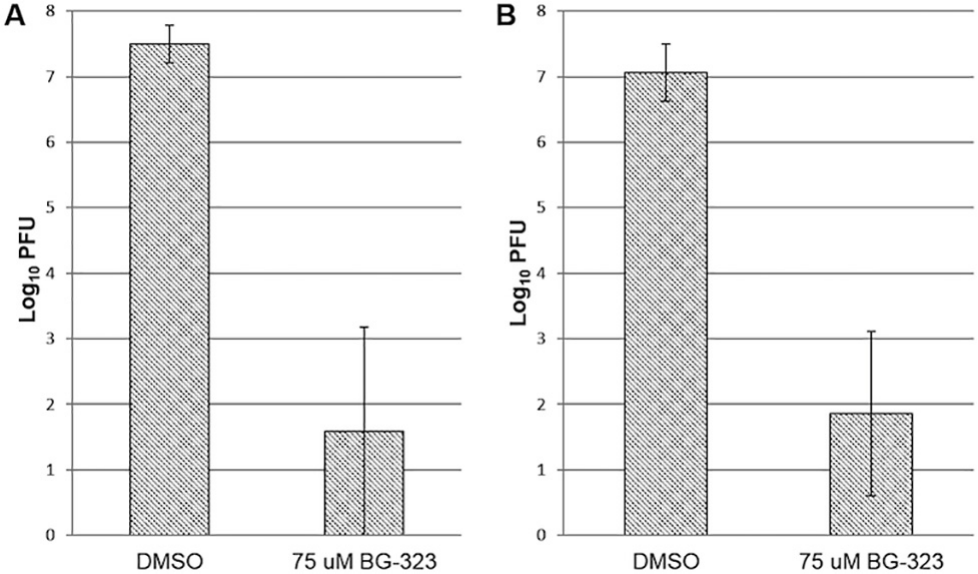
Effects of BG-323 administration on virulent Powassan and West Nile viruses in cell culture. (A) BHK21 cells were infected with 100 PFU Powassan virus (strain LB) and administered either DMSO vehicle or 75 μM BG-323. After 72 hours, media was collected and viral loads were titered using a standard plaque assay (n = 3). (B) BHK21 cells were infected with 100 PFU WNV (NY99) and either DMSO vehicle or 75 μM BG-323. After 72 hours, media was collected and viral loads were titered using a standard plaque assay (n = 3). 75 μM BG-323 caused an approximate 5-fold reduction in viral titers compared to DMSO controls in both WNV and Powassan virus-infected cell cultures. BG-323 treated samples were significantly different than DMSO treated samples by two-tailed student t-test for both viruses with p-values of 0.02 and 0.01, respectively.

We previously tested the efficacy of BG-323 against BSL-2 level West Nile virus (subtype Kunjin) and Yellow fever viruses in cell culture and saw that the compound resulted in approximately 2 and 1 log reduction of titers at 75 μM, respectively. We next sought to examine the antiviral effect of BG-323 against virulent West Nile (NY99 strain) and Powassan (LB strain) viruses in BSL-3 cell culture. BHK21 cells were infected with 100 PFU of West Nile or Powassan viruses and 75 μM BG-323 or DMSO was added at time of infection. Samples were collected 72 hr post infection and titered using standard plaque assays. Treatment with 75 μM BG-323 reduced both West Nile virus and Powassan virus titers by approximately 5 logs (Fig 1A and 1B respectively), indicating that BG-323 is somewhat more active against a virulent mosquito and tick-borne flaviviruses.

### Testing resistance emergence to BG-323

**Fig 2:**
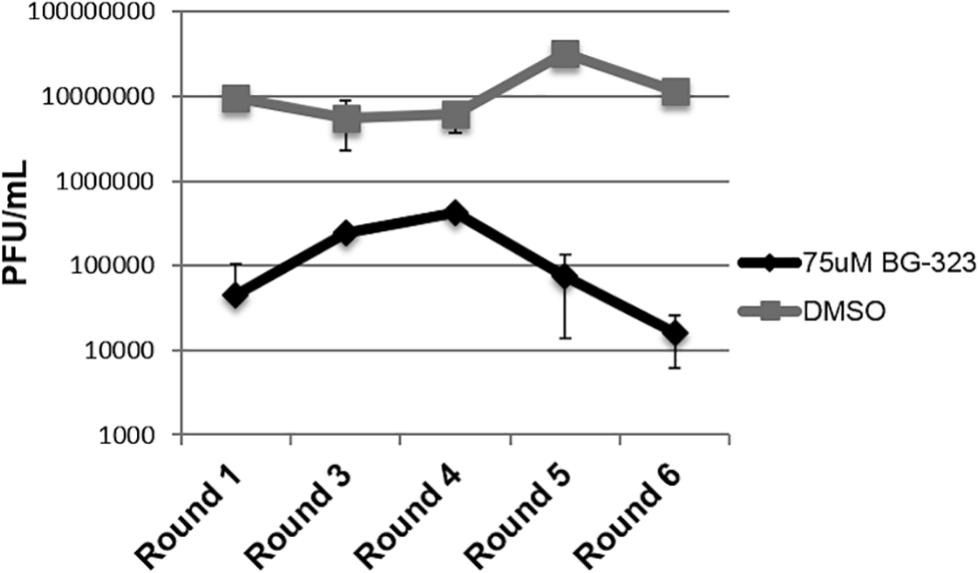
Determination of West Nile virus resistance to BG-323 in cell culture. BHK21 cells were concurrently infected with WNV (Kunjin) and treated with indicated concentrations of BG-323. Assays were performed in triplicate and media with 75 μM of BG-323 was replaced daily. On day 3 post-infection, samples were collected and viral titers were assessed by plaque assay. 100 PFU of virus from each round was used to initiate the next round, for a total of six rounds.

Drug resistance is a major problem in the development of antivirals against RNA viruses. To determine whether flaviviruses could become resistant to BG-323 during long-term exposure to the drug during infection, we monitored how growing Kunjin virus in the presence of BG-323 for extended periods affects viral titers. We infected BHK cells with 100 PFU of Kunjin virus in the presence of 75 μM BG-323 or DMSO, and replenished the media with fresh BG-323 or DMSO daily for 3 days. On day 3 we collected viral samples and determined the titer. We used 100 PFU of virus from the titered samples to infect fresh BHK21 cells and initiated another round of infection. This strategy was repeated for a total of six rounds. We observed that there was no significant increase in viral titers over time in the presence of BG-323 (Fig 2), indicating that during the course of the experiment that resistant mutants did not arise. While this does not rule out the possibility that resistance can arise with longer-term exposure, these data do indicate that resistance to BG-323 is not easily achieved.

### Determination of BG-323 toxicity in mice

**Fig 3:**
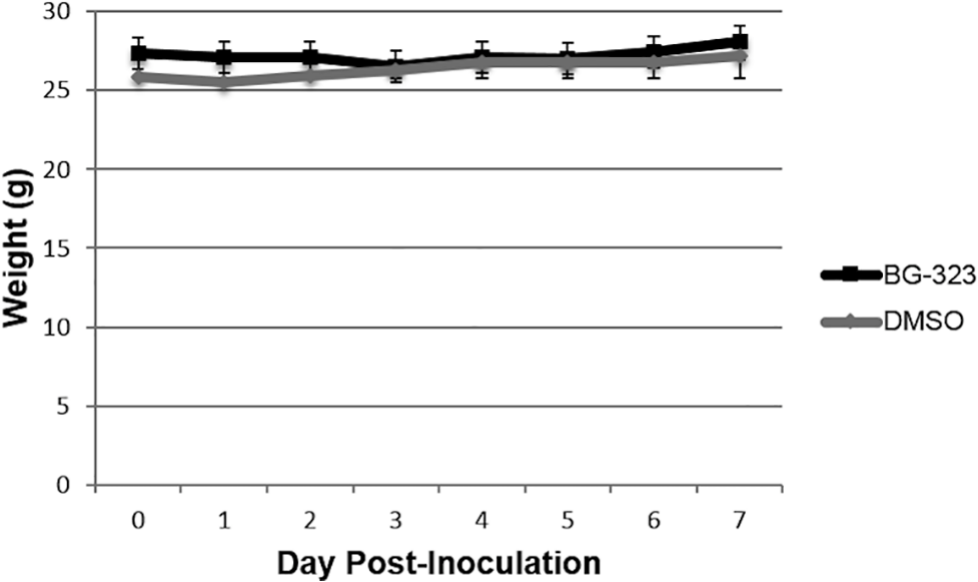
Effects of BG-323 administration on mouse weight. 50 mg/kg BG-323 or DMSO were administered intraperitoneally (IP) to mice and weights were monitored for 7 days in order to determine any gross toxic effects of BG-323.

Based on our *in vitro* antiviral data, we decided to perform *in vivo* testing of BG-323 using a West Nile virus mouse model. Because BG-323 has not been previously administered to animals, we first determined whether the molecule displayed any gross toxicity in animals. 6–8 week ICR mice were treated with 50 mg/kg BG-323 and weights were monitored for seven days. Maximum weight loss percentage was calculated for each mouse. Mouse weights remained relatively constant over the seven-day monitoring period (Fig 3). Though BG-323-treated mice showed greater average weight loss percentage (3.5%) than control mice (1.5%) the differences in weight loss were not significant according to a Mann-Whitney *U* test. Therefore, BG-323 does not appear to be grossly toxic in mice.

### Ability of BG-323 to inhibit West Nile virus in mice

**Fig 4:**
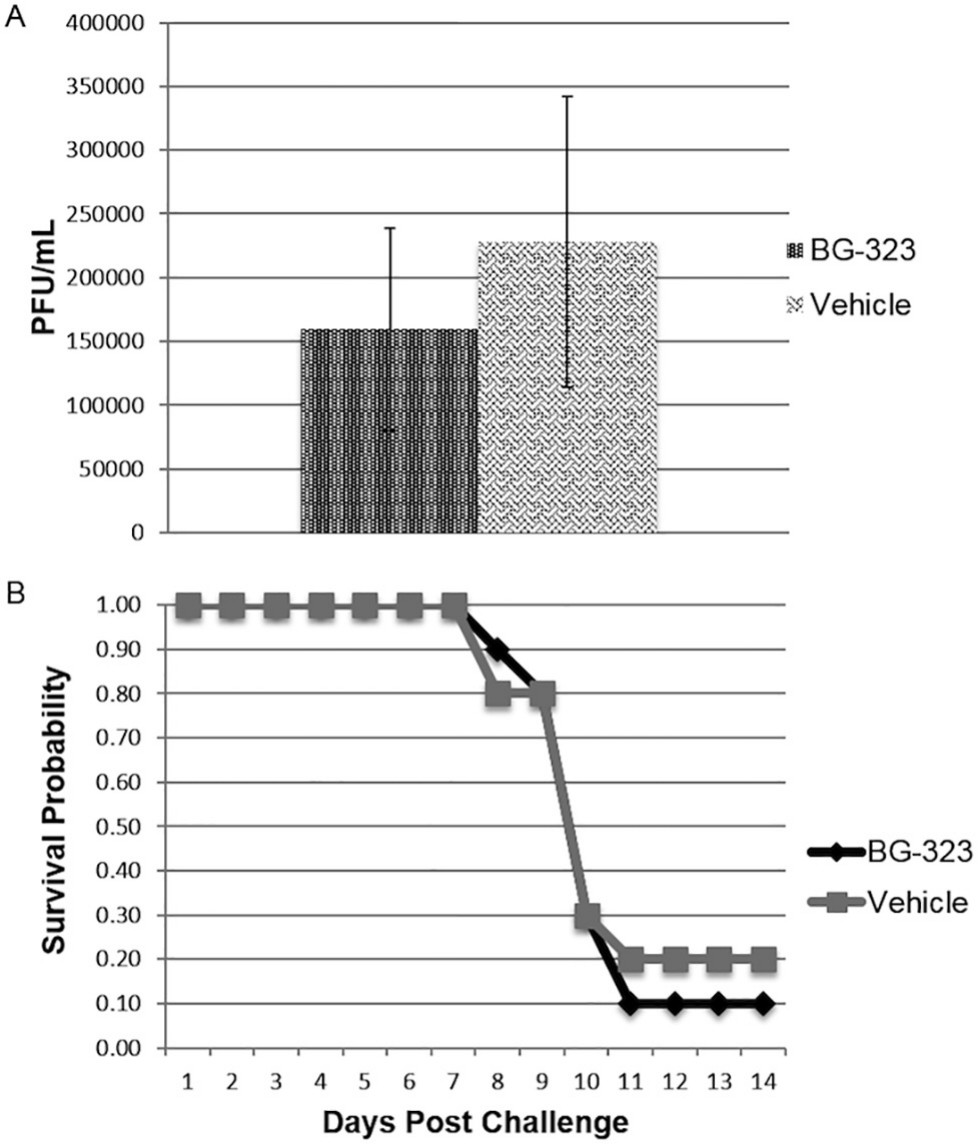
Efficacy of BG-323 in West Nile virus-challenged mice. Mice were challenged with 500 PFU WNV (NY99) via footpad injection then administered 50 mg/kg BG-323 or vehicle by IP inoculation BID for 5 days. Mice were bled on day 3 post challenge and average viral titers were calculated by plaque assay. (A) Average PFU/mL on day three post-challenge are presented for BG-323-challenged and control animals. (B) Clinical scores were assigned animals up to 14 days-post-challenge and Kaplan-Meier estimates of survival probability were calculated.

In order to assess the ability of small molecule BG-323 to inhibit West Nile virus NY99 replication *in vivo*, two groups of 10 mice each were challenged with 500 PFU of West Nile Virus (NY99) via footpad inoculation. Immediately post-challenge, mice were either treated with 50 mg/kg BG-323 or DMSO vehicle by IP inoculation BID for 5 days post challenge. Mice were bled on day three to determine viral titers and clinical scores were assigned to mice up to 14 days post challenge. The average viral titers resulting from sera isolated on day three were 1.6x10^5^ PFU/mL and 2.28x10^5^ PFU/mL for BG-323-treated and control mice respectively (Fig 4A) revealing no significant difference in viral replication between the two groups according to a student t-test and Kaplan-Meier survival probability calculations. These data indicate that survival probability is approximately the same between the two groups (Fig 4B). During assignment of clinical scores, no mice from either group developed signs of disease until day six post-challenge (Table 1).

**Table 1:** Clinical scores of mice treated with BG-323.

*Clinical Scores*	*Days Post-Challenge*													
*Mouse*	*1*	*2*	*3*	*4*	*5*	*6*	*7*	*8*	*9*	*10*	*11*	*12*	*13*	*14*
BG323 1	0	0	0	0	0	1	1	1	2	4				
BG323 2	0	0	0	0	0	1	1	1	2	4				
BG323 3	0	0	0	0	0	1	1	1	4					
BG323 4	0	0	0	0	0	1	1	1	4					
BG323 5	0	0	0	0	0	1	1	1	1	1	1	0	0	0
BG323 6	0	0	0	0	0	1	4							
BG323 7	0	0	0	0	0	1	1	1	4					
BG323 8	0	0	0	0	0	1	1	1	4					
BG323 9	0	0	0	0	0	1	1	1	4					
BG323 10	0	0	0	0	0	1	1	4						
Control 1	0	0	0	0	0	1	4							
Control 2	0	0	0	0	0	1	1	1	4					
Control 3	0	0	0	0	0	1	1	1	4					
Control 4	0	0	0	0	0	1	1	1	1	4				
Control 5	0	0	0	0	0	1	1	1	1	1	1	0	0	0
Control 6	0	0	0	0	0	1	1	1	4					
Control 7	0	0	0	0	0	1	4							
Control 8	0	0	0	0	0	1	1	1	4					
Control 9	0	0	0	0	0	1	1	1	1	1	0	0	0	0
Control 10	0	0	0	0	0	1	1	1	4					

BG-323-challenged and control mice were assigned clinical scores post challenge with West Nile virus NY99 for up to 14 days. Meaning of numerical assignments are as follows: 0 is normal; 1 is questionable illness; 2 is distinct mild illness; 3 is distinct disease; 4 is serious disease/moribund. If a mouse was assigned a clinical score of 4, it was euthanized.

### Determination of BG-323 half-life in mice

**Fig 5:**
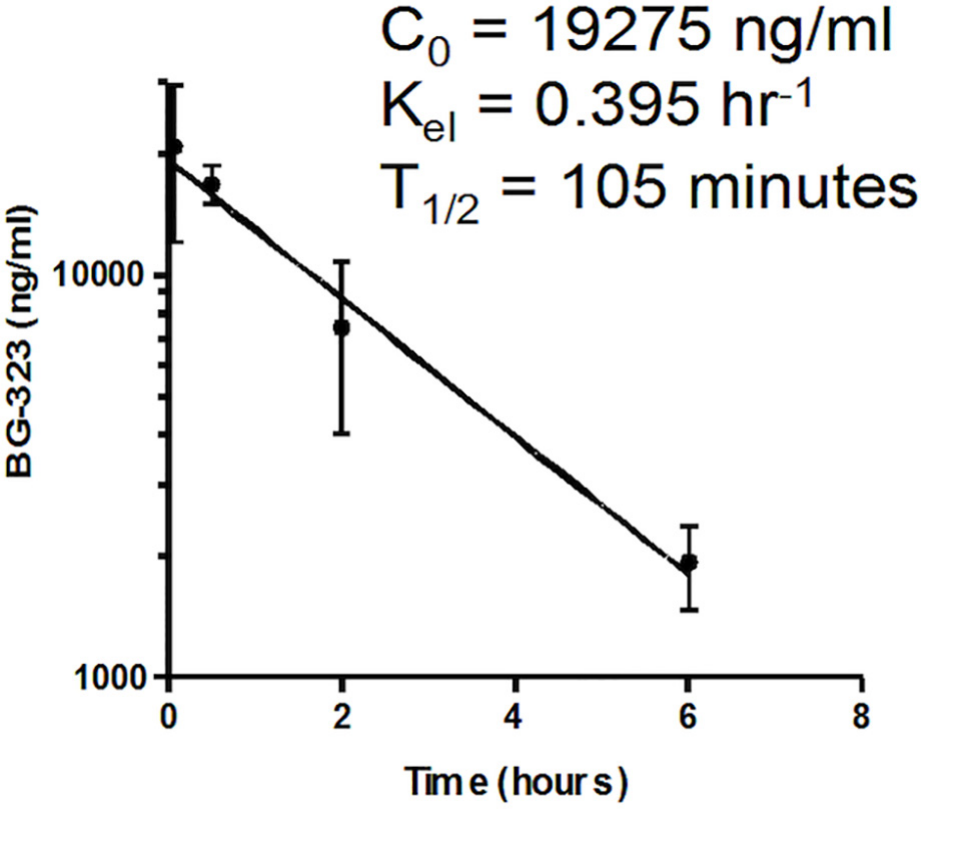
Half-life of BG-323 in mice. BG-323 was administered to mice in a 0.38 mL bolus of 5 mg/mL BG-323. Mice were bled at the indicated times and LC-MS/MS was performed on resulting sera to quantify the concentration of BG-323 present in each sample. Initial concentration (C_0_) was 19275 ng/mL, elimination rate constant (K_el_) was 0.395 hr^-1^, and T_1/2_ was found to be 105 minutes.

To begin to understand why BG-323 was ineffective *in vivo*, we first sought to determine the half-life of BG-323 in mice. 13 mice were dosed with BG-323 (5 mg/mL in a total volume of 0.38 mL), mice were weighed then anesthetized and bled by cardiac puncture 5, 30, 120, and 360 min after BG-323 administration. Isolated sera were analyzed by LC-MS/MS and BG-323 concentrations at each time point were quantified. Five min after administration of BG-323, average serum concentrations reached nearly 21,000 ng/mL and steadily declined over each subsequent time period. The calculated T_1/2_ of BG-323 was found to be 105 min (Fig 5).

### *In vitro* Pharmacokinetic evaluation of BG-323

While we determined that the T_1/2_ of BG-323 in mouse sera was relatively short, there are other potential factors that could also affect efficacy, including cell permeability, metabolic stability, interaction with CYP isoforms, and binding to plasma proteins. In order to ascertain the degree of BG-323 cell permeability, we quantified the amount of BG-323 able to cross Caco-2 epithelial cell monolayers in the apical to basolateral (A to B) and basolateral to apical (B to A) directions. Bidirectional percent recovery and P_app_ were calculated as well as efflux ratio. The percent recovery of BG-323 from A to B and B to A were 43% and 57% respectively and P_app_ from A to B and B to A were 49.4±1 x 10^−6^ cm/s and 49.6±9.5 x 10^−6^ cm/s, respectively (Table 2). Efflux ratio of BG-323 was found to be 1:1, thereby classifying the compound as highly cell permeable with insignificant efflux. These data suggest that BG-323 is cell permeable *in vitro* and is able to enter cells to inhibit viral replication.

**Table 2:** BG-323 Caco-2 cell permeability.

Test Compound	Direction	Percent Recovery	Average P_app_	Efflux Ratio	Permeability Classification	SignificantEfflux
BG-323	A to B	43	49.4±1	1.1	High	NO
	B to A	57	49.6±9.5			

Cell permeability of BG-323 was tested in a Caco-2 cell permeability assay. Direction of BG-323 movement, percent recovery, average apparent permeability (P_app_), efflux ratio, permeability classification and whether observed efflux was classified as significant are indicated.

**Fig 6:**
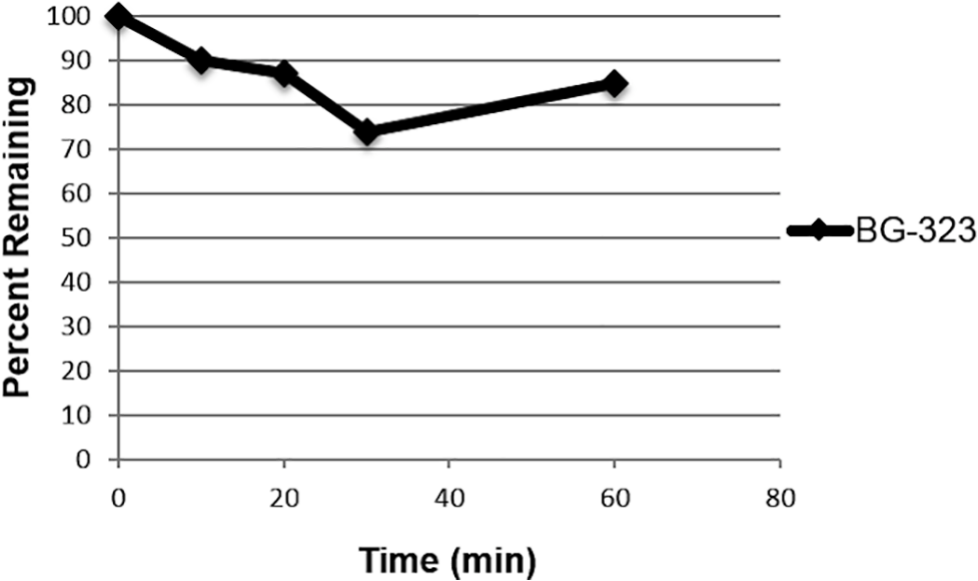
Metabolic stability in human liver microsomes. BG-323 was incubated with pooled human liver microsomes. After reaction initiation with NADPH, reactions were sampled at times 0, 10, 30 and 60 minutes. BG-323 concentrations were determined by LC-MS/MS at each time point (n = 1).

Degradation of BG-323 *in vivo* may account for its relatively short T_1/2_ and lack of *in vivo* antiviral efficacy. Metabolic stability of BG-323 in human liver microsomes was tested to determine whether BG-323 would be metabolized and inactivated by liver enzymes. BG-323 was incubated with human liver microsomal protein and NADPH and reactions were sampled at different time points. BG-323 concentration was quantified at each time point by LC-MS/MS. Percent of BG-323 remaining was calculated for time points 0, 10, 20, 30 and 60 min. Half-life in min as well as *in vitro* intrinsic clearance (CL_int_) were calculated. At time 0, BG-323 concentration was 100% of initial as expected and concentrations steadily decreased over time (Fig 6). At the 60 minute time point 85% of the initial concentration remained and T_1/2_ was calculated to be >60 min. CL_int_ was determined to be <0.023 mL/min/mg protein. These data indicate that BG-323 can be rapidly metabolized by liver enzymes, which may contribute to BG-323s relatively short half-life observed *in vivo*.

**Fig 7:**
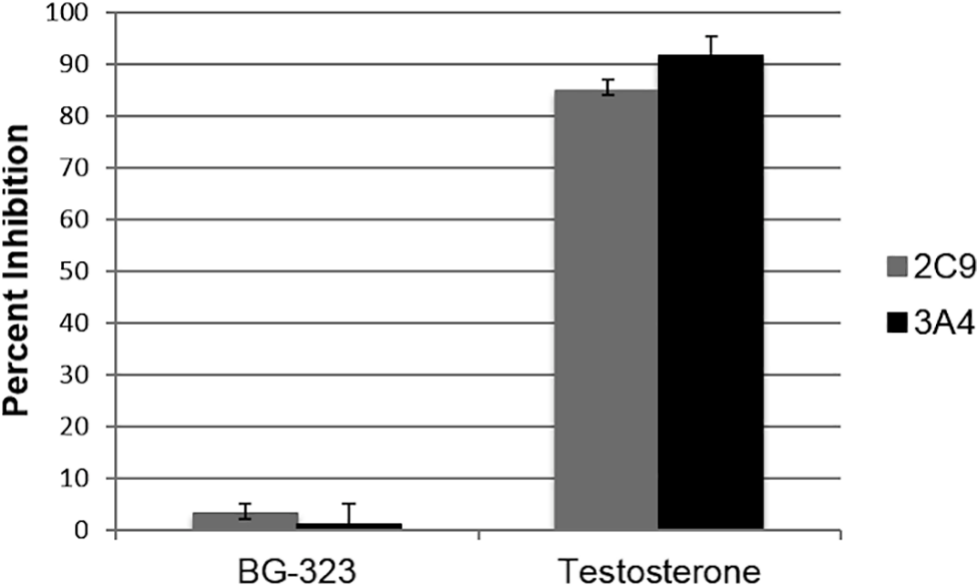
Binding to CYP P450. BG-323 and testosterone were incubated with pooled human liver microsomes. CYP P450 isoform specific substrates and NADPH were added to the reaction. The concentration for each CYP P450 specific substrate metabolite was determined using LC-MS/MS.

In order to ascertain whether BG-323 is metabolized by CYP P450 enzymes in the liver, degree of BG-323 binding to two common CYP P450 isoforms, 2C9 and 3A4, was assessed and compared to the binding of the testosterone internal control. Percent of BG-323 and isoform-specific positive control compound remaining after reaction and percent inhibition for BG-323 and positive control were determined (Fig 7). Binding of BG-323 to CYP isoforms 2C9 and 3A4 was low with the majority of BG-323 remaining unbound post reaction and percent inhibition of the two isoforms in the presence of BG-323 was insignificant compared to positive controls, indicating that CYP P450 interaction is likely not significant *in vivo*, and other metabolic pathways are involved in BG-323 degradation.

**Fig 8:**
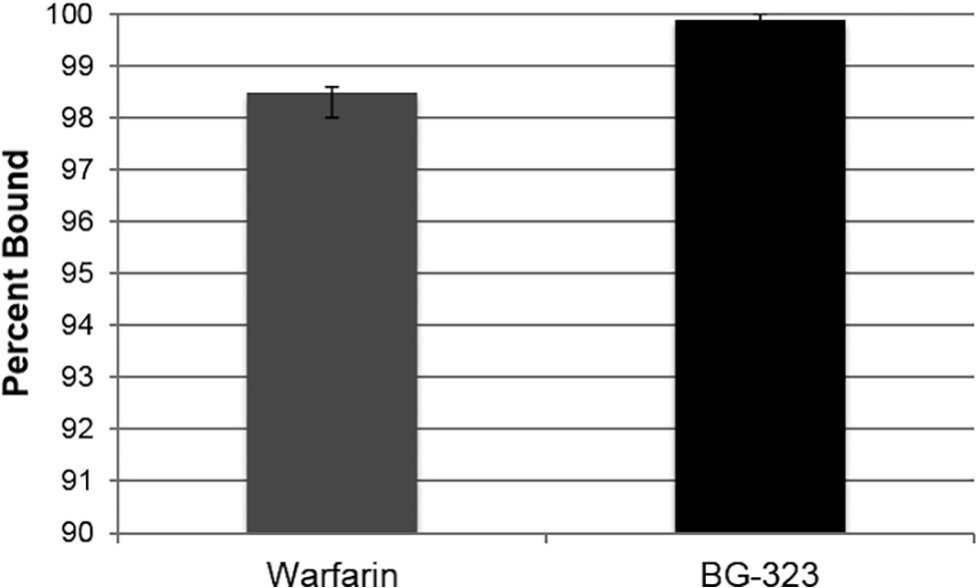
Binding of BG-323 to human plasma proteins. In order to determine the extent to which BG-323 binding human plasma proteins, BG-323 was diluted in plasma and allowed to equilibrate for four hours at 37°C in a Rapid Equilibrium Device. LC-MS/MS was used to quantify the concentration of unbound BG-323 after equilibration. Percent of BG-323 and Warfarin control bound to plasma protein was then calculated.

Finally, we determined whether BG-323 binds human plasma proteins, which would reduce the effective soluble concentration in sera. In order to determine the degree to which BG-323 binds to human plasma binding proteins, BG-323 was diluted in human plasma and was allowed to equilibrate for four hr at 37°C between two sides of a membrane in a Rapid Equilibrium Device. LC-MS/MS was used to quantify the concentration of unbound BG-323 after equilibration. BG-323 was found to be 99.9% bound to plasma proteins after 4 hr compared to the drug standard Warfarin, which was 98.5% bound, indicating that the vast majority of the compound is likely protein bound and may not be able to interact with NS5 in infected cells to block viral RNA replication (Fig 8).

## Discussion

We have previously described the ability of small molecule BG-323 to inhibit flaviviral NS5 capping enzyme activity and the ability of BG-323 to inhibit both West Nile virus (Kunjin subtype) and yellow fever virus. This report describes the continued characterization of the potential antiviral molecule BG-323. We demonstrate here that BG-323 has antiviral efficacy against both virulent mosquito-borne (West Nile NY99) and tick-borne (Powassan) flaviviruses in cell culture and that viral resistance to BG-323 is not easily generated in cell culture. *In vivo* toxicity testing of BG-323 indicates that the molecule is not grossly toxic in mice, but little antiviral effect was observed in mouse models. Further pharmacokinetic analysis suggests that the lack of antiviral efficacy may be due to rapid inactivation of BG-323 through liver metabolism and sequestration of the small molecule due to human plasma protein binding.

Inherent within the lead optimization process is the need to evaluate the likely fate of a drug after dosing. Metabolic stability of a small molecule is inversely related to the potential drug’s rate of clearance and half-life, which in turn determine how often a drug must be administered in order to keep concentrations at therapeutic levels. Our data indicate that while BG-323 is able to inhibit viral replication *in vitro*, the metabolic stability of the small molecule *in vivo* is suboptimal. This is likely due to the biotransformation of BG-323 as the small molecule is modified or degraded by metabolic enzymes. Strategies to circumvent metabolic destabilization of drug-like compounds include blocking the active metabolic site(s) in the small molecule by replacing a highly reactive group with a less reactive group [27,28], adding a bulky group that increases steric hindrance proximal to the reactive group [29], incorporating the labile group into a ring structure [30] or removing the labile group from the small molecule altogether [31]. Such alterations can lead to a several fold increase in the stability of a small molecule and can significantly increase the half-life of the compound. Other successful strategies for increasing metabolic stability rely on the specificity of metabolic enzymes for their substrates. Changing chirality [32], reducing lipophilicity [33] and changing the size of cyclized groups within the molecule [30] have also been shown to have a favorable effect on small molecule stability. The application of one or more of these strategies to BG-323 could stabilize the compound sufficiently to increase the half-life and potentially increase the ability of the BG-323 to inhibit viral replication *in vivo*.

Another potential contributor to the lack of BG-323 efficacy in the mouse model could be the high percent of BG-323 binding to human plasma proteins. Small molecule plasma protein binding can have the effect of sequestering the drug, reducing penetration of the drug into the target tissue and ultimately limiting interaction with the intended therapeutic target [32]. However, it is important to note that while not optimal, high binding of a small molecule to plasma proteins does not necessarily mean the compound is not druglike. In fact, a study of 1500 frequently prescribed drugs revealed that 43% are >90% bound by human plasma binding proteins [34]. In addition, there are several structural modification strategies with can ameliorate the human plasma binding tendencies of a potential drug candidate and contribute to greater bioavailability. Quantitative structure-activity relationships looking at the qualities of small molecules that are associated with binding to plasma proteins have determined that, in order of high to low effect, reducing lipophilicity, reducing acidity, increasing basicity, reducing nonpolar area and increasing polar surface area are all sound strategies for decreasing the overall concentration of drug bound to human plasma proteins [35]. Structurally modifying groups in BG-323 to take advantage of these strategies could increase the percentage of BG-323 that is available to inhibit NS5 capping enzyme activity and flaviviral replication *in vivo*.

In addition to structurally modifying BG-323 in order to optimize its pharmacokinetic profile, it is possible to formulate the small molecule with excipients that confer favorable properties. Incorporation of BG-323 with a surfactant, lipid-based delivery system or complexing BG-323 with molecules such as cyclodextrins would serve to enhance stability and may also have the added benefits of increasing solubility of the small molecule [36,37,38,39]. This strategy would serve to increase bioavailability, allow dispersal of BG-323 to occur more rapidly and reduce the impact of short compound half-life on overall antiviral efficacy.

## Conclusion

We have shown here that small molecule BG-323 is able to inhibit replication of virulent WNV and tick-borne Powassan virus *in vitro* and that continued application of BG-323 does not elicit signs of drug resistance in WNV. Further, the small molecule is not grossly toxic but does not display a significant anti-flaviviral efficacy *in vivo*, which is likely due to the relatively short half-life and high percentage of binding to human plasma proteins. Future studies with BG-323 will employ structure-based and formulation strategies to attempt to ameliorate the effects of plasma protein binding and enhance compound stability and half-life *in vivo*.
